# Temporal fluctuations in chemotaxis gain implement a simulated-tempering strategy for efficient navigation in complex environments

**DOI:** 10.1016/j.isci.2021.102796

**Published:** 2021-06-28

**Authors:** Omer Karin, Uri Alon

**Affiliations:** 1 Department Molecular Cell Biology, Weizmann Institute of Science, Rehovot, Israel; 2 Wellcome Trust–Cancer Research UK Gurdon Institute, University of Cambridge, Cambridge, UK; 3 Department of Applied Mathematics and Theoretical Physics, Centre for Mathematical Sciences, University of Cambridge, Cambridge, UK

**Keywords:** Biological sciences, Microbiology, Bioinformatics, Mathematical biosciences, Systems biology

## Abstract

Bacterial chemotaxis is a major testing ground for systems biology, including the role of fluctuations and individual variation. Individual bacteria vary in their tumbling frequency and adaptation time. Recently, large cell-cell variation was also discovered in chemotaxis gain, which determines the sensitivity of the tumbling rate to attractant gradients. Variation in gain is puzzling, because low gain impairs chemotactic velocity. Here, we provide a functional explanation for gain variation by establishing a formal analogy between chemotaxis and algorithms for sampling probability distributions. We show that temporal fluctuations in gain implement simulated tempering, which allows sampling of attractant distributions with many local peaks. Periods of high gain allow bacteria to detect and climb gradients quickly, and periods of low gain allow them to move to new peaks. Gain fluctuations thus allow bacteria to thrive in complex environments, and more generally they may play an important functional role for organism navigation.

## Highlights

• High chemotaxis gain impairs patch crossing, whereas low gain impairs peak localization
• Stochastic switching between low and high gain periods provides a superior strategy
• Stochastic strategy is analogous to simulated tempering sampling algorithm
• Simulated tempering may be a widespread principle for efficient navigation

## Graphical abstract

**Figure:**
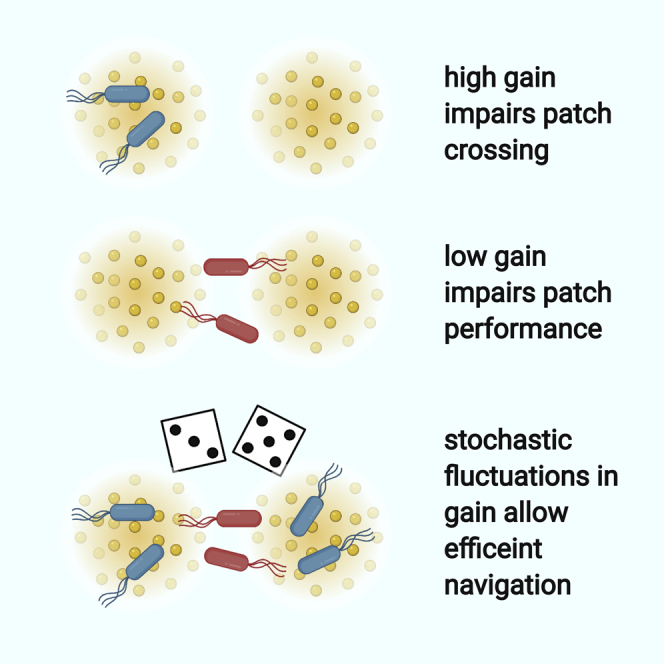

## Introduction

**Figure 1:**
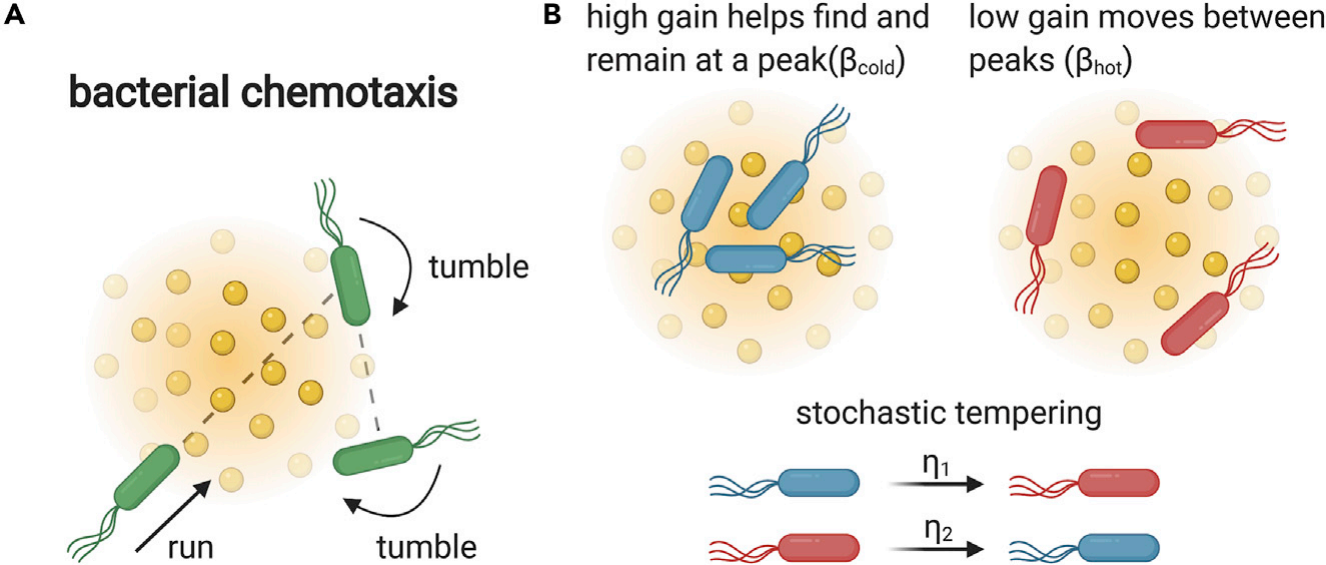
Stochastic heterogeneity in pathway gain in bacterial chemotaxis (A) Bacterial chemotaxis is the navigation process by which bacteria accumulate near peaks of chemoattractants and away from chemorepellants. Chemotaxis is performed by adjusting the tumbling rate according to the sensed concentration of the ligand, so that bacteria tumble less when they move up attractant gradients or down repellant gradients. (B) The accumulation of bacteria around attractant peaks is determined by an inverse temperature parameter β, which is proportional to the chemotaxis gain, also called pathway sensitivity. Recent experiments showed that there is large and persistent non-genetic heterogeneity in gain, suggesting that there may be temporal fluctuations in β (Salek et al., 2019). These fluctuations may be short lasting or long lasting (e.g., due to cell-to-cell variation in receptor copy numbers). The functional significance of these fluctuations is currently unclear.

Bacteria navigate up and down gradients of chemicals in a process called chemotaxis. Chemotaxis is achieved by transitions between swimming at a roughly uniform speed and constant direction (“runs”) and random reorientations (“tumbles”). Bacteria climb gradients of chemical ligands by modulating their tumbling frequency, so that tumbling rate decreases when the bacteria move up gradients of chemoattractants and increases when they go down the gradient (Figure 1A).

Chemotaxis is well-characterized in terms of its molecular signaling circuit. The circuit implements a non-linear integral feedback loop, which brings tumbling rates precisely back to the baseline after changes in input (exact adaptation) and allows the bacteria to respond to relative changes in ligand input (fold-change detection [FCD]) across a wide dynamic range (Shoval et al., 2010; Lazova et al., 2011). Analogous navigation systems also appear in eukaryotes (Polin et al., 2009; Arrieta et al., 2017) and simple animals (Larsch et al., 2015; Borba et al., 2020).

Several aspects of the chemotaxis phenotype are highly variable between individual isogenic bacteria (reviewed by Waite et al. [2018]; Colin and Sourjik [2017]). This variability results, at least in part, from stochastic fluctuations in the abundance of proteins in the chemotaxis signaling pathway (Spudich and Koshland, 1976; Levin et al., 1998) and from tunable post-translational modifications (Kamino et al., 2020) and is subject to genetic control.

A key question is whether phenotypic variability has an adaptive functional role for chemotaxis. Most of the research on the adaptive role of variability has focused on the baseline tumbling rate (Spudich and Koshland, 1976; Park et al., 2010; Matthäus et al., 2009; Frankel et al., 2014; Dufour et al., 2016; Keegstra et al., 2017). Some individual cells show high tumbling and others show low tumbling rate, and this behavior lasts for an entire cell generation. In each individual cell, the adaptation time to a step attractant change is inversely proportional to that cell's tumbling frequency (Spudich and Koshland, 1976). A recent study also demonstrated that some cells have discrete on/off temporal fluctuations in the chemotaxis signaling pathway, with a timescale of minutes (Keegstra et al., 2017). In an important theoretical study, Frankel et al. proposed that high tumbling rate is preferable when the navigation goal is near, whereas low tumbling rate is preferable when the goal is far (Frankel et al., 2014). This prediction was confirmed by subsequent experimental work (Dufour et al., 2016; Waite et al., 2016), which also suggested that heterogeneity can be beneficial for amplifying the contribution of high-performing individuals at the tail of the phenotype distribution. Stochastic fluctuations in tumbling rate may also contribute to efficient exploration of the environment by generating Lévy walk search behavior (Tu and Grinstein, 2005; Matthäus et al., 2009).

Recently, another major source of cell-cell variability was discovered, whose functional role is still not clear. This is variability in the *pathway gain* (Salek et al., 2019). The gain, also called sensitivity, determines how strongly bacteria change their tumbling rate in response to a given gradient (Figure 1B) (Sourjik and Berg, 2002; Colin and Sourjik, 2017). Salek et al. (2019) found that gain varies between genetically identical individual cells by approximately an order of magnitude. Cell-cell variation in pathway gain is surprising, because large gain increases both chemotaxis efficiency and how tightly bacteria accumulate around attractant peaks (Salek et al., 2019). One may therefore expect that gain should be maximized. It has been proposed that cell-cell variability in pathway gain may be beneficial for bet-hedging by preventing the accumulation of too many bacteria in a small spatial region (Salek et al., 2019).

Here we consider the possibility that the observed cell-cell variability in gain is due to temporal fluctuations, which can last for less or more than the generation time of an individual cell.

We propose a functional role for temporal fluctuations in pathway gain using a formal analogy to sampling algorithms in physics and computer science. Gain fluctuations allow bacteria to efficiently navigate in complex environments where the ligand distribution has many local maxima and minima. Without gain fluctuations, bacteria would get stuck at local maxima. To show this, we establish a mapping between bacterial chemotaxis and a widely used method from statistical physics for sampling complex probability distributions known as *simulated tempering,* which uses dynamic changes in temperature to cross energy barriers (Swendsen and Wang, 1986; Marinari and Parisi, 1992; Hukushima and Nemoto, 1996; Earl and Deem, 2005; Lee et al., 2018). We show that stochastic fluctuations in pathway gain provide a biological implementation of simulated tempering and demonstrate that it allows colonization and growth in complex, patchy environments.

Our model makes minimal assumptions and can be readily generalized to FCD-based navigation systems in other organisms. We therefore conclude by discussing several possible generalizations of our results.

## Results

### Model for bacterial chemotaxis as a sampling process

We begin by establishing a connection between chemotaxis and the sampling of a probability distribution, where the distribution is the distribution of chemoattractant in space. The sampling framework we use is the Langevin Monte Carlo (LMC) approach. LMC is widely used for efficient sampling (Roberts and Tweedie, 1996; Neal, 2011; Girolami and Calderhead, 2011; Dalalyan, 2014) and for global optimization (Chiang et al., 1987; Gelfand and Mitter, 1991; Lee et al., 2018; Erdogdu et al., 2018; Ma et al., 2019; Chen et al., 2020). In this article, we exploit the analogy between chemotaxis and LMC to understand the efficiency of chemotaxis from an algorithmic perspective.

To establish the relation between bacterial chemotaxis and LMC sampling, we use the chemotaxis model of Tu et al. (2008). This model provides good agreement with experimentally measured responses of bacteria to temporally varying stimuli (Shimizu et al., 2010) and has become a standard model for chemotaxis (Menolascina et al., 2017; Salek et al., 2019; Alon, 2019). The model describes the fast (sub-second) inhibition of the tumbling rate *λ(t)* by the attractant ligand input *L(t)*. It also describes the slower (seconds-minutes) adaptation to a step change in *L(t)* that is due to receptor methylation, *m(t),* a negative feedback process. The tumbling rate is controlled by a fast internal “receptor activity” variable *a(t)*:

(Equation 1) $$a\left(t\right)=\frac{1}{1+e^{N\left(\alpha \left(1-m\right)+\log\left(1+\frac{L}{K_{I}}\right)-\log\left(1+\frac{L}{K_{A}}\right)\right)}}\approx \frac{1}{1+e^{N\left(A\left(1-m\right)+\log\left(\frac{L}{K_{I}}\right)\right)}}$$

where $K_{I},K_{A},\alpha ,N\;$are constants and the approximation holds in the range of receptor sensing: $K_{I}\ll L\ll K_{A}$. The dynamics of the slow negative feedback process is given by:

(Equation 2) $$\dot{m}\left(t\right)=\omega \left(1-\frac{a}{a_{0}}\right)$$

where $a_{0}$ is the steady-state level of a and ω sets the timescale of adaptation. The tumbling rate is given by:

(Equation 3) $$\lambda \left(a\right)=\frac{1}{\tau }{\left(\frac{a}{a_{0}}\right)}^{H}$$

where τ is steady-state run time, which is on the order of 1 s. Typical values for the constants $\alpha ,N,a_{0},K_{I},K_{A},\omega ,H$ are provided in Table S1. The adaptation time of m, which is approximately inversely proportional to $\left(1-a_{0}\right)N\alpha \omega$ (STAR Methods), is on the order of seconds to minutes.

The model has several important features. It has the FCD property, where tumble responses to a time-varying input *L(t)* depend only on changes of *L(t)* relative to its baseline (Shoval et al., 2010; Shimizu et al., 2010; Adler and Alon, 2018). More generally, a(t) is proportional to the logarithmic derivative of the low-frequency signals in the input, with a frequency threshold that is inversely proportional to the adaptation time. These features, which are common in sensory circuits (Adler and Alon, 2018), have been experimentally demonstrated (Lazova et al., 2011). Thus, a bacteria running in a static ligand field *L* in direction $\vec{u}$ relative to the gradient will have $a\left(t\right)\propto v\vec{u}\cdot \nabla \log\left[L\right]\left(x\right)$, where v is the swimming speed of the bacteria.

The run-and-tumble movement of *E. coli* is nearly isotropic at time and length scales much larger than τ,vτ (Berg, 1993). This movement is thus well approximated by a diffusion process with diffusion constant $D=\frac{v^{2}d^{-1}}{z_{\theta }+\tau^{-1}}$, where *d* is the dimension and $z_{\theta }$ is rotational diffusivity, which is small compared with the tumbling frequency $\tau^{-1}$. The temporal evolution of the bacterial population can therefore be modeled by a Fokker-Planck equation that depends on the aforementioned signaling dynamics, as shown by several studies that also provide excellent correspondence to empirical distributions with plausible parameters (Si et al., 2012; Dufour et al., 2014; Menolascina et al., 2017). Here we analyze the equivalent Langevin equation, which describes the stochastic time evolution of the location of an individual bacterium:

(Equation 4) $$dx=\text{χ }\nabla \log\;L\left(x\right)dt+\sqrt{2D}\;dW$$

where W is a 1-dimensional (1D) Wiener process and the advection parameter χ is proportional to the swimming speed squared: $\text{χ}=\left[{\text{v}}^{2}\tau^{-1}/{\left(z_{\theta }+\tau^{-1}\right)}^{2}\right]HN\left(1-a_{0}\right)$ (Si et al., 2012). Note again that Equation 4 applies in the range where sensing is logarithmic $K_{I}\ll L\ll K_{A}$.

The advection parameter χ is also called the chemotactic sensitivity coefficient (Salek et al., 2019). We will study the 1D case (d = 1), but our conclusions generalize to 2 and 3 dimensions. We will also neglect the fact that the run direction after tumble may be correlated with the previous run direction, which increases diffusivity but does not affect our conclusions.

Equation 4 is equivalent to a continuous LMC process that samples an invariant probability distribution P(x): at long times, the bacterial density converges to P(x). This distribution is proportional to a power β of the ligand distribution (Lee et al., 2018):

(Equation 5) $$P\left(x\right)\propto e^{\beta \;\log\;L\left(x\right)}=L{\left(x\right)}^{\beta }$$

where:

(Equation 6) $$\text{β}=\frac{\text{χ}}{D}=\frac{\tau^{-1}}{z_{\theta }+\tau^{-1}}HN\left(1-a_{0}\right)\approx HN\left(1-a_{0}\right)$$

Equation 5 is due to the log-sensing property of chemotaxis, which results in an advection term that is proportional to the logarithmic gradient in Equation 4. The logarithmic gradient is equivalent to the advection term of LMC. A search strategy that does not respond to the logarithmic gradient will therefore not be equivalent to LMC.

The parameter β, which in statistical physics is proportional to the inverse temperature, determines the degree to which the invariant distribution is concentrated around the peaks of the attractant profile L(x). For our purposes, it is important to note that the “inverse temperature” β is proportional to the chemotaxis gain, as well as to the baseline receptor activity ${\text{a}}_{0}$ (Equation 6). As there are temporal fluctuations in ${\text{a}}_{0}$ (Keegstra et al., 2017) and potentially also in chemotaxis gain, there may be large temporal fluctuations in β. Remarkably, β does not depend sensitively on swimming speed v or average run duration τ, which may be environment dependent; it depends only on intracellular signaling parameters.

When the inverse temperature parameter β is very small (β≪1), the bacterial distribution P(X) is nearly uniform on its support; when it is very large (β≫1), P(X) is concentrated around the global maximum of attractant L(x); and when β=1, the process samples the attractant distribution precisely, P(x)∼ L(x).

**Figure 2:**
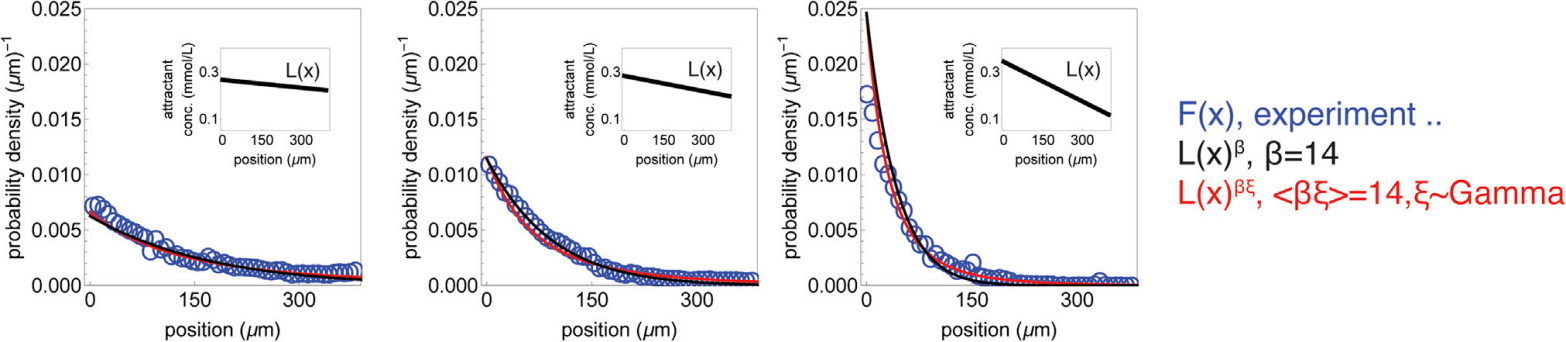
Inference of the inverse temperature parameter β from chemotaxis in linear gradients Kalinin et al. (2009) performed experiments where a bacteria *(E. coli)* navigated linear gradients *L(x)* of the chemoattractant MeAsp in a microfluidic device (*insets*). The accumulation profiles *F(x)* (blue circles) correspond well to an invariant probability distribution proportional to $L{\left(x\right)}^{\beta }$ where β=14 (black lines). This correspondence holds even if we account for the heterogeneity in β observed in Salek et al., by taking the invariant probability distribution to be proportional to $L{\left(x\right)}^{\xi \beta },\xi ∼Gamma\left[2.25,\;35\right]$ (red lines).

What is the typical value of β for *E. coli* chemotaxis? Microfluidics experiments in a linear gradient in the sensing range (Kalinin et al., 2009) show that bacteria converge to a population distribution of $P\left(X\right)\propto L{\left(x\right)}^{\beta }$ where β∼14 (Figure 2, black lines). The analytical estimate from Equation 4 with the parameters provided in Si et al. (2012) also gives a high value of β∼27. The discrepancy may be partially due to *a* being away from the adapted state *a*_*0*_ during gradient climbing (see Hu and Tu [2014] for details). Both estimates, however, suggest that *E. coli* tends strongly toward optimization, in the sense that cells converge to a distribution that is tightly concentrated around the attractant peak.

The experiments of Salek et al. suggest that there is large cell-cell variation in the pathway gain HN (and hence in β) (Salek et al., 2019). Accounting for this variation does not hinder the fit to the empirical distributions (Figure 2, red lines) and can explain the long tail observed in the steep linear gradient (Figure 2, right panel). However, the variance in pathway gain measured by Salek et al. suggests large cell-cell variation in β (i.e., a 95% range of 2<β<37 for the estimate β≈14). This raises the question of what is the role of the large variability of β.

### A constant gain would prevent efficient chemotaxis

**Figure 3:**
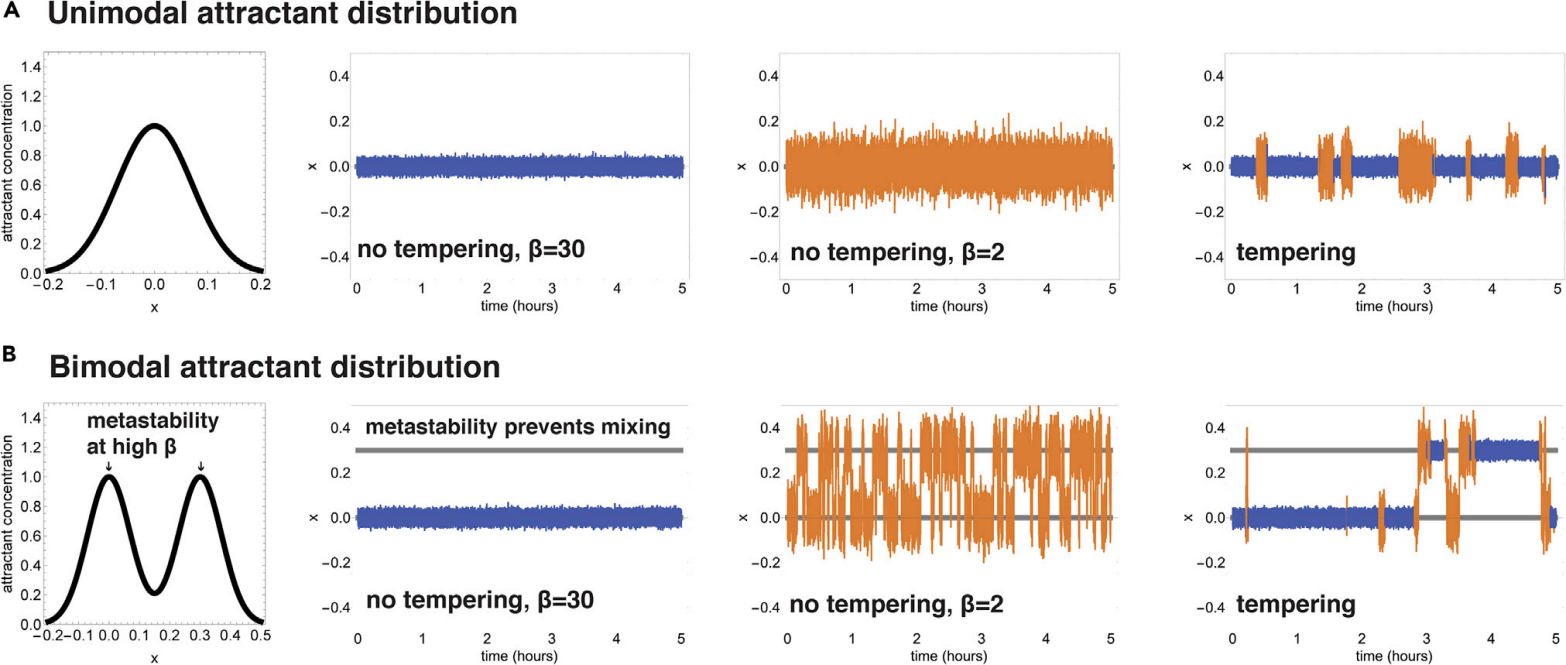
Stochastic tempering allows efficient movement between attractant peaks in multi-modal attractant distributions (A and B) We simulated the chemotaxis Langevin processes in either unimodal (A) or bimodal (B) attractant distributions. The processes had a high gain, parametrized by an “inverse temperature” parameter β (β=30, blue, left panel); a low gain (β=2, orange, middle panel); or a tempering strategy with stochastic switches between high and low gain (β=30–β=2). Parameters for all processes are provided in the STAR Methods section. Although the high-β process localizes well around the unimodal peak, it fails to cross between peaks in the bimodal case. The low-β process, on the other hand, crosses well between the peaks but localizes poorly around each peak. The tempering strategy can both localize well around each peak (in periods here β is high) and cross well between the peaks in periods where β is low.

Understanding the adaptive importance of a trait requires us to understand how this trait contributes to the ability of the organism to survive and reproduce. For bacterial chemotaxis, an effective chemotaxis strategy is considered to be one that has rapid and tight accumulation of bacterial populations around peaks of attractants (or away from repellants) (Clark and Grant, 2005; Celani and Vergassola, 2010). In the simple case of a single-peaked Gaussian patch of attractant (Figure 3A), this is clearly optimized by maximizing pathway gain (and therefore maximizing “inverse temperature” β), because this maximizes the velocity in the direction of the gradient, as well as the tightness of the invariant distribution around the peak.

Such single-peaked patches are unlikely, however, to fully represent the natural environments in which *E. coli* live. We will therefore consider a more general environment that contains multiple attractant peaks, namely, a multimodal attractant distribution (Figure 3B). In such complex environments, the bacteria face two important tasks. The first is to navigate toward a maximum of attractant, and the second is to colonize many attractant peaks. As we will show using the analogy with sampling by LMC, these tasks are incompatible with simply maximizing or minimizing β.

The fundamental problem of sampling in complex multimodal environments is that of metastability—becoming stuck at one peak for long times. Consider an environment *L(x)* that consists of two Gaussians of unit width with means at x=0,x=2μ (this corresponds, for example, to patches of dissolved organic matter). If a sampling process that samples $L{\left(x\right)}^{\beta }$ starts around the first peak x=0, when will it visit the second peak? This transit time will be limited by the probability density around the midpoint (or valley) between the peaks x=μ, which is proportional to $e^{-\frac{1}{2}\beta \mu^{2}}$. Therefore, the time it takes to cross between modes increases exponentially with inverse temperature β and with the distance μ. The feasibility of exploring multiple peaks is thus extremely sensitive to the choice of β and its relation with the statistical properties of the environment. Similar considerations apply for linear gradients from steady sources that decay like L(x)=(1/x), where the probability density at distance μ goes like $\mu^{-\beta }$ (Figure S1). One possibility to avoid metastability is to set β low enough to allow transitions between the peaks. This, however, may be an unfavorable solution, because it comes at the cost of worse localization near the peaks: the bacterial distribution is spread out and nearly uniform.

To summarize, a chemotactic strategy with a single (or narrowly distributed) β faces an inauspicious trade-off. Choosing a large inverse temperature β results in tight accumulation around the attractant peak, but crossing between patches becomes intractable; a low β may allow efficient crossing between patches, but the bacteria will not accumulate around the peaks of the attractant distribution.

### Tempering allows efficient navigation in complex environments

To address this problem, we propose that bacteria employ a tempering strategy with stochastic temporal changes in inverse temperature β. This provides a physiological function to the stochastic variation in gain, which proportionally affects β. Our inspiration is the simulated tempering approach to LMC, in which stochastic switches between temperatures allow sampling of complex, multi-modal distributions (Marinari and Parisi, 1992; Lyubartsev et al., 1992). When multiple instances are run in parallel, the method is known as parallel tempering (Hansmann, 1997; Earl and Deem, 2005). Simulated tempering is also related to the simulated annealing method for global optimization (Kirkpatrick et al., 1983; Van Laarhoven and Aarts, 1987), in which temperature is changed over time according to a defined schedule.

For simplicity, we analyze stochastic switches between two inverse temperatures: $\beta_{hot},\beta_{cold}$, with transition rates: $\eta_{1}:\beta_{cold}\to \;\beta_{hot},\;\eta_{2}:\beta_{hot}\to \;\beta_{cold}$, similar to the binary fluctuations observed by Keegstra et al. (2017) (our results generalize to stochastic switches between many temperatures). Bacteria spend on average a fraction $\varepsilon =\left(\eta_{1}/\eta_{1}+\eta_{2}\right)$ of the time at $\beta_{hot}$ and (1−ε) of the time at $\beta_{cold}.$ Intuitively, this strategy can be efficient because crossing valleys between peaks becomes feasible (because a bacterium spends a fraction ε of the time at $\beta_{hot}$, where it can overcome potential barriers), and it can also localize efficiently around each peak when at $\beta_{cold}$.

To quantitatively test the importance of tempering, we will analyze two scenarios: colonization of multiple patches and escape from an unfavorable patch.

**Figure 4:**
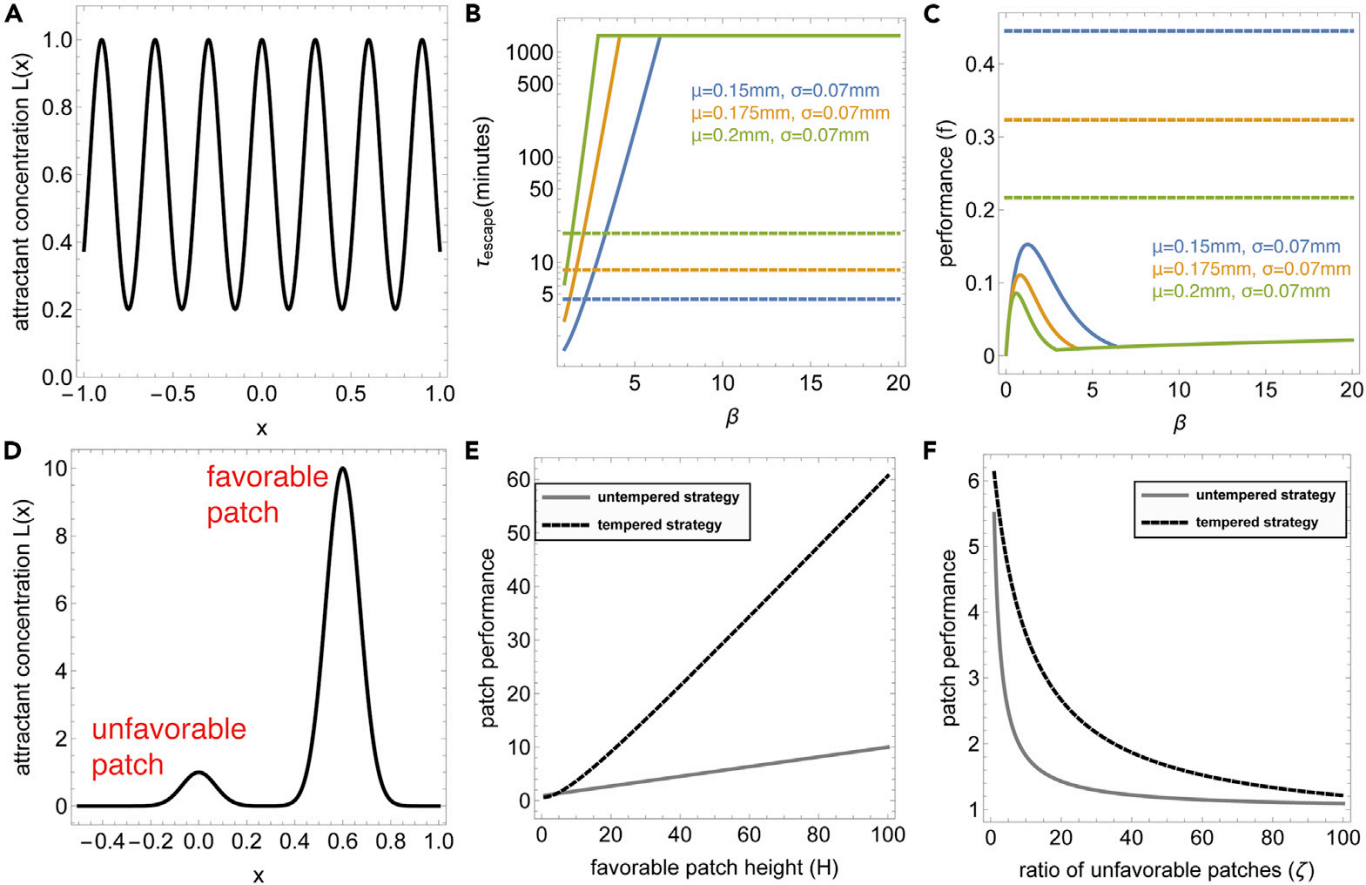
Stochastic tempering provides a superior strategy for patch colonization in multi-modal environments (A) The serial patch model is an infinite mixture of Gaussian attractant patches with standard deviation σ and maxima placed at …−4μ,−2μ,0,2μ,4μ,….. Bacteria start at x=0. (B) Time to cross over to an adjacent patch $\tau_{escape}$ increases exponentially with β for the untempered strategy (solid lines, plotted for various μ). For the tempered strategy crossing time is determined by $\beta_{hot}$ (dashed lines, plotted for $\beta_{hot}=1,\beta_{cold}=20,\varepsilon =\frac{1}{3},D=0.0009\;mm^{2}/sec$). We assume a patch lifetime of $\tau_{patch}=1day$. (C) Performance *f* for the untempered strategy as a function of β (solid lines) decays to zero at low and high values of β due to the trade-off between colonization and localization. The untempered strategy outperforms it by decoupling colonization and localization (dashed lines). (D) The asymmetric patch model is an infinite mixture of small unfavorable Gaussian patches (height h=1) and large favorable Gaussian patches (height h=H≫1). For panels (E and F) we denote by ζ the ratio between unfavorable and favorable patches. (E) Expected value of patch performance (normalized by$\sqrt{\beta_{cold}}$ or $\sqrt{\beta }$) for tempered (black) and untempered (gray) strategies for various values of *H* setting ζ=10 (other parameters are as in other simulations). (F) Normalized expected value of patch performance as a function of ζ, setting H=10.

For the first scenario, which we call the *serial patch model*, we will consider an environment that consists of a 1D array of Gaussians with standard deviation σ and with maxima placed at $\mu_{n}=2n\mu ,\;n\in Z$: $L_{n}\left(x\right)=e^{-\frac{1}{2\sigma^{2}}{\left(x-2n\mu \right)}^{2}\;}$ (Figure 4A). The midway points between patches are at distance μ from each peak, which is large compared with the standard deviation μ/σ≫1 (i.e., the patches are separated). We assume that the performance of an individual bacteria (or its lineage), f, depends on the product of how many patches the bacteria visited $f_{v}$ and its performance over each patch $f_{p}$, that is, $f=f_{v}f_{p}$. The migration of bacteria in this model is limited by metastability at the midway points. The crossing rate between patches can therefore be estimated by the Kramers approximation (Kramers, 1940):

(Equation 7) $$\tau_{escape}^{-1}\approx \frac{\sqrt{-U^{\prime \prime }\left(0\right)U^{\prime \prime }\left(\mu \right)}}{2\pi }e^{-\frac{U\left(\mu \right)-U\left(0\right)}{D}}\approx \frac{\text{βD}\sqrt{\mu^{2}-\sigma^{2}}}{2\pi \sigma^{3}}2^{\beta }e^{-\frac{\mu^{2}}{2\sigma^{2}}\beta }$$

where U is the potential function $U=-\text{χ}\log\left(e^{-\frac{1}{2}{\left(x-2\mu \right)}^{2}}+e^{-\frac{1}{2}x^{2}}\right)$ and χ=βD. In Figure 4B we plot the value of $\tau_{escape}$ for various values of μ,σ and β . One can see that the crossing rate $\tau_{escape}^{-1}$ is very sensitive to the choice of β (see also Figure S2 for estimation of $\tau_{escape}$ for a wide range of μ,σ and β).

As $\tau_{escape}^{-1}$ is the rate-limiting step for the colonization of new patches, we can model the colonization process as random walk where the bacteria have probability $p=\tau_{escape}^{-1}$ to move to each of the adjacent patches. The number of patches visited up to time t (divided by $\sqrt{t}$) will be on the order of the mean displacement:

(Equation 8) $$f_{v}\approx \sqrt{2p}=\sqrt{\frac{\text{βD}\sqrt{\mu^{2}-\sigma^{2}}}{\pi }}2^{\frac{\beta }{2}}e^{-\frac{\mu^{2}}{4\sigma^{2}}\beta }$$

Note that aforementioned equation can be generalized for patches with various separation distances (due to different variance or mean position) by taking the geometric mean of the values of $\tau_{escape}^{-1}$.

For the performance in each patch, we consider a performance metric that depends on localization around the peak: the overlap integral between the location of the bacteria P(x) and the attractant peak of L(x) (δ≪1):

(Equation 9) $$f_{p}=\frac{\int_{-\delta }^{\delta }P\left(x\right)dx}{\int_{-\delta }^{\delta }L\left(x\right)dx}=\frac{\int_{-\delta }^{\delta }\sqrt{\frac{\beta }{2\pi \sigma^{2}}}e^{-\frac{\beta }{2\sigma^{2}}x^{2}}dx}{\int_{-\delta }^{\delta }\sqrt{\frac{1}{2\pi \sigma^{2}}}e^{-\frac{1}{2\sigma^{2}}x^{2}}dx}\approx \sqrt{\beta }$$

We can also consider other performance metrics—the important aspect is that β improves localization around the peak, and thus improves $f_{p}$. The overall performance is therefore:

(Equation 10) $$f=f_{p}f_{v}=\beta \sqrt{\frac{\text{D}\sqrt{\mu^{2}-\sigma^{2}}}{\pi }}2^{\frac{\beta }{2}}e^{-\frac{\mu^{2}}{4\sigma^{2}}\beta }$$

The performance rises and then falls as a function of β (Figure 4C, solid lines).

The equation for f reveals the fundamental trade-off between colonization and localization—as β is important for localization but detrimental for colonization, it is impossible to optimize both $f_{p}$ and $f_{v}$ at the same time. Moreover, the optimal β is highly sensitive to μ (which is contingent on the environment), and a choice of β that is too low or too high results in poor performance.

Now consider a tempered strategy with stochastic switches between $\beta_{hot},\beta_{cold}$. We make the conservative assumption that the time the bacteria spends at $\beta_{cold}$ makes no contribution to escaping from patches, and thus:

(Equation 11) $$\tau_{escape}^{-1}\left(\mu \right)\approx \varepsilon \frac{{\text{β}}_{\text{hot}}\text{Dμ}}{2\pi \sigma^{3}}2^{{\text{β}}_{\text{hot}}}e^{-\frac{\mu^{2}}{2\sigma^{2}}{\text{β}}_{\text{hot}}}$$

This holds as long as the bacteria spends enough time in ${\text{β}}_{\text{hot}}$ to escape (i.e., $\eta_{2}$ is smaller than $\tau_{escape}^{-1}$). Thus:

(Equation 12) $$f_{v}\approx \sqrt{\varepsilon \frac{{\text{β}}_{\text{hot}}\text{D}\sqrt{\mu^{2}-\sigma^{2}}}{\pi }}2^{\frac{{\text{β}}_{\text{hot}}}{2}}e^{-\frac{\mu^{2}}{4\sigma^{2}}{\text{β}}_{\text{hot}}}$$

We also make the conservative assumption that the time the bacteria spends at $\beta_{hot}$ makes no contribution to patch performance, and thus patch performance is:

(Equation 13) $$f_{p}\approx \left(1-\varepsilon \right)\sqrt{\beta_{cold}}$$

### The overall performance is

(Equation 14) $$f=f_{p}f_{v}=\left(1-\varepsilon \right)\sqrt{\varepsilon }\sqrt{\beta_{cold}}\sqrt{\frac{{\text{β}}_{\text{hot}}\text{D}\sqrt{\mu^{2}-\sigma^{2}}}{\pi }}2^{\frac{{\text{β}}_{\text{hot}}}{2}}e^{-\frac{\mu^{2}}{4\sigma^{2}}{\text{β}}_{\text{hot}}}$$

We can see a clear difference between the performance of the tempered strategy (Equation 14) and the untempered strategy (Equation 10)— the tempered strategy allows one to optimize separately $f_{p}$ (by increasing $\beta_{cold}$) and $f_{v}$ (by decreasing $\beta_{hot})$ at a fixed cost of the pre-factor $\left(1-\varepsilon \right)\sqrt{\varepsilon }$. Therefore, choosing a low $\beta_{hot}$ and a high $\beta_{cold}$ (independent of μ) can easily outperform the untempered strategy (Figure 4C, dashed lines). Tempering therefore allows for efficient balancing of colonization and patch utilization.

The second scenario is the *asymmetric patch model*, which explores the problem of global optimization. Consider two Gaussian patches with standard deviation σ and with maxima placed at 0,2μ, and with very different heights h=1,h=H, so H≫1 (Figure 4D). We assume that the performance increases proportionally to patch height (which corresponds to attractant concentration). We can compute the transition rate from the unfavorable patch (h=1) to the favorable patch (h=H):

(Equation 15) $$\tau_{escape}^{-1}\approx \frac{\text{βD}\sqrt{h\mu^{2}-\frac{1}{2}{\left(1+h\right)}^{2}\sigma^{2}}}{\left(1+h\right)\pi \sigma^{3}}{\left(1+h\right)}^{\beta }e^{-\frac{\mu^{2}}{2\sigma^{2}}\beta }$$

The exponential dependence of the escape rate on β again means that for well-separated patches (large μ/σ) it is unfeasible to leave the unfavorable patch when β is large. As the bacteria can in principle get trapped in either of the patches, the expected value for the performance is $f_{p}\approx \frac{1}{2}\left(H+1\right)\sqrt{\beta }$.

For the tempered strategy, on the other hand, the bacteria can cross between the patches in the $\beta_{hot}$ state and localize effectively in the $\beta_{cold}$ state. From Equation 5, the relative time it spends at the favorable patch is $1/\left(1+H^{-\beta_{hot}}\right)$ (we again assume that patch crossing occurs only in the $\beta_{hot}$ state), so the performance becomes proportional: $f_{p}=\left(1-\varepsilon \right)\left(\frac{H}{1+H^{-\beta_{hot}}}+\frac{1}{1+H^{\beta_{hot}}}\right)\sqrt{\beta_{cold}}$. Taking, for example, $\beta =\beta_{cold},\beta_{hot}=1$, and H≫1, we find $f_{p}\approx \left(1-\varepsilon \right)H\sqrt{\beta_{cold}}$. Thus, for two patches, the tempered strategy is better when the cost of tempering ε is smaller than 1/2.

What about a more general case, where there are only a few favorable patches $n_{h=H}$ among many unfavorable patches $n_{h=1}$ (i.e. $\frac{n_{h=1}}{n_{h=H}}=\zeta \gg 1$)? The untempered strategy has a performance of $\frac{f_{p}}{\sqrt{\beta_{cold}}}=\frac{1}{\zeta^{-1}+1}+\frac{1}{\zeta +1}H\to 1$, because it is unlikely that the starting patch will be a favorable one. On the other hand, for the tempered strategy, we have $\frac{f_{p}}{\sqrt{\beta_{cold}}}=\left(1-\varepsilon \right)\left(\frac{1}{\zeta^{-1}H+1}+\frac{H}{\zeta H^{-1}+1}\right)=\left(1-\varepsilon \right)\frac{H^{2}+\zeta }{H+\zeta }$. This performance depends on the ratio of H/ζ—a very favorable patch (H≫ζ≫1) can yield high performance $\frac{f_{p}}{\sqrt{\beta_{cold}}}=\left(1-\varepsilon \right)H$ even when there are many unfavorable patches in the environment. Tempering therefore allows for efficient utilization of favorable patches in the presence of other (potentially many) unfavorable patches (Figures 4E and 4F).

In conclusion, these models suggest that the tempered strategy can yield superior performance in the presence of multiple attractant patches.

This quantitative theory was developed for static attractant patches. In reality, patches have a finite lifetime $\tau_{patch}\;$due to their consumption, diffusion/advection, or environmental effects. The timescale $\tau_{patch}$ is variable—for marine sources, for example, it can range from seconds to minutes for patches of dissolved organic matter to hours to days for more persistent sources such as marine aggregates or phytoplankton (Kiørboe et al., 2002; Stocker et al., 2008). Patch lifetime can be incorporated into the model. It sets a maximal value for $\tau_{escape}$: $\tau_{escape}<\tau_{patch}$. Given this value, it is possible to determine whether the cost of tempering (the prefactor of Equation 14) indeed outweighs the cost of using an untempered strategy, with the escape rate now capped by $\tau_{patch}.$ In Figures 4B and 4C we set $\tau_{patch}=1day$, and in Figure S3 we plotted $\tau_{escape}$ and f taking $\tau_{patch}=10\;min$ and $\tau_{patch}=1\;week$.

### Tempering provides efficient growth and colonization in complex environments

**Figure 5:**
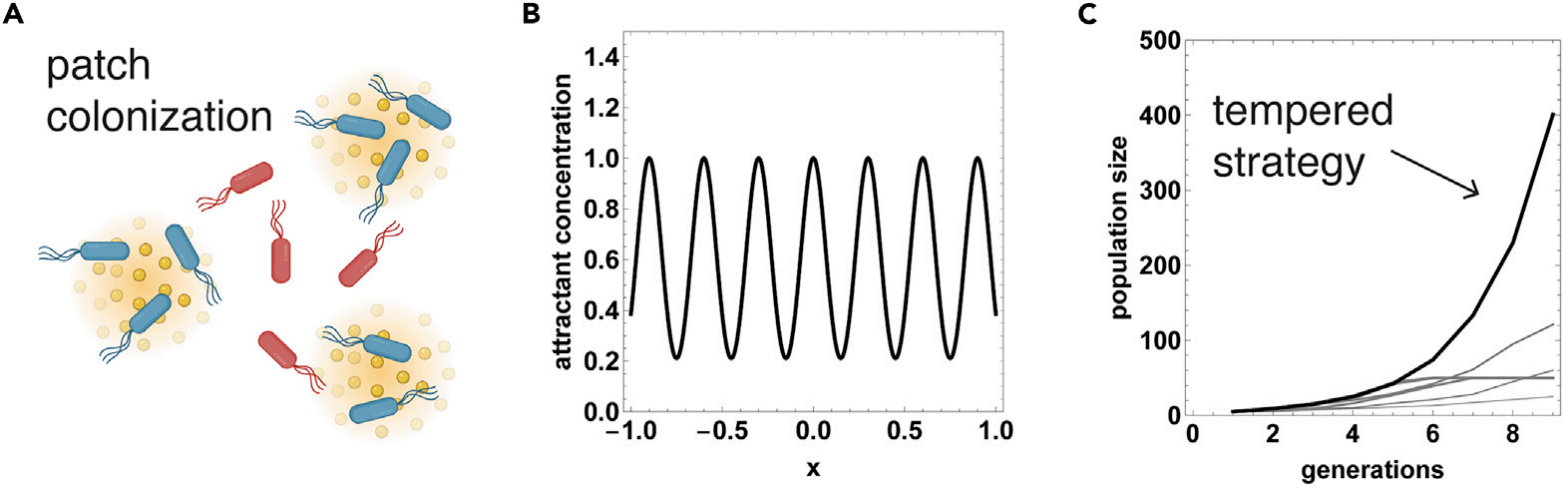
Tempering improves growth and colonization of multi-modal environments in population simulations (A) A simple model of patch colonization, where bacteria migrate and colonize attractant patches. Each simulated bacteria performs chemotaxis. At the end of each generation-interval, the bacteria replicates if local attractant concentration is higher than $l_{rep}$, and if the patch is occupied by less than *K* bacteria. Here $l_{rep}=0.95$ and *K = 50*. Red bacteria represent the $\beta_{hot}$ state, whereas blue bacteria represent the $\beta_{cold}$ state. (B) Simulations for a 1D case where the attractant patches are Gaussians with height = 1 and standard deviation σ*= 0.07* mm (see Figure S3 for other parameter choices). G = 21 patches are placed at μ= *0.15* mm distance from each other. (C) Constant-β (constant gain) strategies (gray lines, thicker lines correspond to higher β, namely, β=2,3.4,5.9,10.1,17.5,30) either fail to localize around peaks (low β) or suffer from metastability and fail to colonize new patches (high β). This results in lower overall population growth. The simulated tempering strategy (black line) localizes well around peaks and can colonize new patches, yielding larger population growth.

In addition to the theoretical analysis of tempering in a patchy environment, we also simulated a minimal model for bacterial population dynamics (Figure 5A). We assume that the environment consists of *G* equal-height Gaussian patches with peaks at 0,2μ,4μ,…,2(G−1)μ, and standard deviation σ (Figure 5B). The bacteria navigate and grow in this environment, such that after every generation-interval ($t_{gen}$) the bacteria replicate if they sense an attractant level higher than a threshold $l_{rep}$ (accumulation near peak). Bacteria start at x = 0. To encourage colonization of new patches, we also assume that each patch has a finite carrying capacity *K*. For simplicity we again assume a static attractant profile; the carrying capacity may then be due to the nutrient depletion, while the attractant is a separate navigational cue that is present at large quantities.

Constant−β strategies perform poorly in such environments (Figure 5C). If β is high, the bacteria localize effectively near the first local maximum of the attractant. They therefore initially grow rapidly; however, the finite carrying capacity *K* of the patch prevents further growth, and, due to metastability, they only very slowly colonize other patches. This results in limited population expansion. For the bacteria to colonize other patches, β needs to be lowered, but this prevents effective localization near the peak and so also compromises growth.

Bacteria with a tempering strategy that temporally switch between high and low β, on the other hand, can effectively grow and colonize (Figure 5C). During the time the bacteria spend at high β, they effectively accumulate at a local peak. Spending time at low β helps the bacteria cross valleys, thus colonizing new patches. This results in superior overall growth rate. Importantly, the strategy is robust to the geometry and size of the attractant peaks—spending some time at a low enough β prevents metastability for diverse environments, even if the average β is very high. As we noted earlier, it is only important that the bacteria spend long enough in $\beta_{hot}$ to allow for patch crossing. As we showed analytically, the choice of $\beta_{hot},\beta_{cold}$ does not need to be tuned for a particular environment, as the simple strategy of switching between high and low β either outperforms or performs similarly to constant-β strategies for diverse environments (Figure S4).

### Theory on LMC sampling can provide insight into chemotaxis strategies

So far, we have shown that bacterial chemotaxis is analogous to LMC-based sampling and used this analogy to demonstrate the importance of stochastic fluctuations in pathway gain for efficient navigation in complex environments. We demonstrated this for an environment that consists of multiple Gaussian attractant patches. We conclude by developing the analogy between patch colonization and sampling more formally, which allows us to generalize our results.

For an attractant ligand distribution L(x) we can define the target probability distribution of the bacteria as $l_{\beta }\left(x\right)\propto L{\left(x\right)}^{\beta }$, which is the invariant distribution of Equation 4. Let $\rho_{t}\left(x\right)$ be the distribution of locations sampled by the bacteria by time *t*. We are interested in how fast $\rho_{t}\left(x\right)$ converges to $l_{\beta }\left(x\right)$. For this we need to measure how different $\rho_{t}\left(x\right)$ is from $l_{\beta }\left(x\right)$. One way to do this is to use the Kullback-Leibler (KL) divergence of $\rho_{t}\left(x\right)$ with respect to $l_{\beta }\left(x\right)$:

(Equation 16) $$H_{l_{\beta }}\left(\rho_{t}\right)=\int \rho_{t}\left(x\right)\log\frac{\rho \left(x\right)}{l_{\beta }\left(x\right)}dx$$

KL divergence is a measure of *relative entropy*. It is non-negative, and minimized $H_{l_{\beta }}\left(\rho_{t}\right)=0$ at $\rho_{t}=l_{\beta }$. There is extensive mathematical literature on the convergence of the KL divergence for Langevin processes (Villani, 2008). An important result is that Langevin dynamics can be viewed as optimization (or steepest descent) of $H_{l_{\beta }}\left(\rho_{t}\right)$ in the space of probability distributions (Jordan et al., 1998; Wibisono, 2018), with dynamics given by:

(Equation 17) $$\frac{dH_{l_{\beta }}\left(\rho_{t}\right)}{dt}=-J_{l_{\beta }}\left(\rho_{t}\right)$$

where $J_{l_{\beta }}$ is the relative Fisher information:

(Equation 18) $$J_{l_{\beta }}\left(\rho_{t}\right)=\int \rho_{t}\left(x\right)\|\nabla \log{\frac{\rho \left(x\right)}{l_{\beta }\left(x\right)}}^{2}dx\|$$

As can be seen from the aforementioned equations, convergence can be rapid if we can bound the KL divergence with the Fisher information:

(Equation 19) $$H_{l_{\beta }}\left(\rho \right)\leq \frac{1}{2\text{Α}}J_{l_{\beta }}\left(\rho \right)$$

which yields:

(Equation 20) $$H_{l_{\beta }}\left(\rho_{t}\right)\leq e^{-2\text{Α}t}H_{l_{\beta }}\left(\rho_{0}\right)$$

That is, the convergence of the KL divergence is exponentially fast. Equation 19 is equivalent to a condition known as the L*ogarithmic Sobolev* I*nequality (LSI)* (Gross, 1975; Bakry and Émery, 1985), with a Sobolev constant Α. LSI holds for strongly log-concave distributions (and in particular for Gaussians)—$l_{\beta }$ satisfies Equation 19 if it is Α-log concave. LSI is also equivalent to a Gaussian-like concentration of $l_{\beta }$ (Ledoux, 1999; Gozlan et al., 2011). We can therefore conclude that chemotaxis (either untempered or tempered) can sample such Gaussian-like attractant patches rapidly.

For multi-modal distributions, such as mixtures of Gaussians, this rapid convergence does not hold, as we have seen the time to cross between modes increases exponentially with their distance for an untempered strategy. However, it is possible to prove that a tempered strategy converges rapidly, at least on a mixture of Gaussians with equal variance (Madras and Randall, 2002; Woodard et al., 2009; Lee et al., 2018). The proof, which is due to Lee et al. (2018), is based on a decomposition theorem by Madras and Randall (2002); informally, it states that if the space can be partitioned into subsets in such a way that the LMC process converges rapidly within each subset, and transitions between subsets occur rapidly enough, then the LMC process converges rapidly overall (STAR Methods). For an environment that consists of a collection of Gaussian attractant patches of equal variance, it is possible to define such a partition (Ge et al., 2017; Lee et al., 2018). If the high-temperature process crosses rapidly between the partition sets (that is, $\beta_{hot}$ is chosen low enough), then the entire process can converge rapidly.

## Discussion

We studied the functional significance of variation in pathway gain in bacterial chemotaxis. Gain variation is puzzling, because individuals with low pathway gain climb gradients poorly and accumulate less tightly around attractant peaks, and thus selection should push towards maximal gain. We considered the possibility that variation is due to (potentially long-lasting) temporal fluctuations. We suggest that such fluctuations in gain are crucial for efficient chemotaxis in complex environments with multiple attractant maxima and minima. This is because a strategy with a single (or tightly distributed) pathway gain either cannot escape local peaks, if gain is high, or localizes poorly around peaks, if gain is low. A variable-gain strategy overcomes both of these problems. This strategy is a biological implementation of the simulated-tempering algorithm used in statistical physics and computer science for sampling complex distributions.

To analyze the function of gain variation, we made a formal analogy between bacterial chemotaxis and the LMC algorithm, a widely used method for sampling probability distributions. Standard LMC often fails to efficiently sample from multi-modal distributions. It gets stuck on local maxima, because the time to cross valleys depends exponentially on the distance between the peaks, and can easily become intractable. Simulated tempering LMC employs a tempering strategy that consists of stochastic switches in the temperature parameter. The tempering strategy makes the crossing of valleys computationally tractable, while sacrificing efficient localization near the peaks for only a fraction of the time. We show that tempering is analogous to stochastic fluctuations in the chemotaxis pathway gain. This allows rapid colonization of new patches by bacteria, while maintaining efficient localization within each patch.

The model presented here is based on the mean-field approximation that averages over the internal state of the bacteria (Si et al., 2012). Although this approximation works well in some parameter regimes (such as shallow gradients), it cannot account for large deviations in internal state that bacteria experience due to positive feedback between run duration and sensing. This was addressed recently in an important theoretical paper (Long et al., 2017). Long et al. showed that the positive feedback between motion and sensing can result in an almost switch-like dynamics, with very long runs when climbing gradients and persistent tumbling when descending the gradient. These dynamics become dominant when the positive feedback timescale $\tau_{E}=\left(L/NHv\right)$ (*L* is the length scale of the gradient) becomes short compared with adaptation time. This effect causes large variability in the internal state of the bacteria (including receptor methylation), which depends on the behavior of the bacteria, and it improves gradient climbing efficiency and accumulation around peaks. Tempering is therefore still required to allow for efficient crossing between peaks.

The present tempering role for temporal fluctuations in gain adds to previous concepts on the functional benefits of phenotypic heterogeneity. Two well-studied concepts are bet-hedging (Kussell and Leibler, 2005; Wolf et al., 2005; Xue and Leibler, 2017; Martín et al., 2019) and division of labor (Ackermann, 2015; Adler et al., 2019). Bet-hedging is defined as diversification that is beneficial for buffering against uncertain changes in the environment. For instance, diverse behavioral strategies help animal populations to overcome different invasion stages and conditions (Sih et al., 2012; Wolf and Weissing, 2012; Carere and Gherardi, 2013; Forkosh et al., 2019). The tempering strategy studied here is beneficial even when the environment is static. In addition, unlike division of labor, which is a population-level property, the tempering strategy can be beneficial at the level of the individual organism, allowing efficient navigation.

It is interesting to consider how tempering is affected by another important source of heterogeneity in bacteria, namely, cell-cell variation in the adaptation time (Spudich and Koshland, 1976). In addition to the functional roles discussed in the introduction, fluctuations in adaptation time can also complement tempering for efficient navigation in complex environments. This is because the bacterial chemotaxis signaling circuit acts as a low-pass filter on the logarithmic derivative of the input (which controls the accumulation around peaks), with a filtering time-window that is proportional to the adaptation time (Tu et al., 2008). Slower adaptation therefore makes the bacteria “see” smoother inputs, which can also accelerate sampling (Ma et al., 2019), again at the cost of poorer localization around local peaks.

In addition to tempering, other behavioral strategies can also help bacteria escape from local attractant peaks. One such strategy is to increase the variation in run duration. It has been proposed that *E. coli* may perform an approximation of a Lévy walk, where the run durations are drawn from a power-law distribution instead of an exponential distribution, due to slow fluctuations in signaling molecules (Tu and Grinstein, 2005; Matthäus et al., 2009). This is supported by a recent study that characterized *E. coli* run-length distributions using 3D tracking (Huo et al., 2021). Mathematically, it is possible to account for the power-law run distribution in the sampling equation by using fractional differentiation, as shown in a recent article by Şimşekli (2017). Lévy walks are effective for searching for randomly distributed, sparse targets (Viswanathan et al., 1999); however they may be suboptimal when the target is close, or in the presence of bias (Palyulin et al., 2014). In principle, a Lévy walk can occasionally generate very long runs that can cross between peaks, while most of the time staying close to the peak. However, there is an important difference between the tempering and Lévy walk strategies. The tempering strategy allows the bacteria to cross between peaks, but bacteria remain attracted to areas of high attractant concentration; in contrast, in the Lévy walk the long runs are random and can send the bacteria far from attractant patches, where the gradient may no longer be detectable. The tempering strategy may therefore be preferable when attractant peaks are dense, whereas the Lévy walk strategy may be preferable when they are sparse.

Although the LMC analogy was developed for bacterial navigation, its underlying assumptions are general and can extend to other organisms that combine FCD input sensing with stochastic navigation. As the benefit from tempering (balancing peak localization with exploration of new peaks) can in principle apply to a wide range of navigation systems, we would expect tempering to be a widespread strategy. One potential example for this is the chemotaxis of *Dictyostelium,* which is based on FCD sensing (Janetopoulos et al., 2004; Kamino and Kondo, 2016; Kamino et al., 2017) and stochastic navigation (Amselem et al., 2012). It will be interesting to test whether this system, which exhibits stochastic fluctuations in signaling components (Arai et al., 2010), also performs tempering. Another potential example is the dopamine system in vertebrates, which controls movement and is based on FCD sensing of expected reward (Tobler et al., 2005; Karin and Alon, 2021). The effective inverse temperature for the dopamine system is β≈1, and it is therefore closer to sampling of rewards rather than optimization, in line with experimental observations on choice behavior across vertebrates (McDowell, 2013). In this system, tempering can be implemented by fluctuations in dopamine gain, which may be due to changes in the level of other neuromodulators such as endogenous opioids (Smith et al., 2011).

The analogy between LMC-based sampling and chemotaxis can help address additional fundamental questions in biological navigation. One important outstanding question is why stochastic navigation is so prevalent across different organisms. The run-and-tumble navigation of bacteria is markedly less efficient in climbing gradients than direct reorientation according to the spatial gradient, because run-and-tumble movement is nearly isotropic and uncorrelated with the gradient (Berg, 1993). It is thought that bacteria use run-and-tumble because they cannot measure spatial gradients along their body, as shown by Berg and Purcell (1977) and Bialek and Setayeshgar (2005) (but see also Thar and Kühl [2003]). However, stochastic navigation is also employed by organisms that can sense spatial gradients along their body, such as *Chlamydomonas* and *C. elegans* (Polin et al., 2009; Luo et al., 2014; Pierce-Shimomura et al., 1999). This raises the question of whether stochastic navigation may be preferable under some conditions to direct spatial-gradient reorientation.

The analogy to sampling and optimization approaches from physics and computer science can provide insight into this question. Stochastic navigation is analogous to LMC-based sampling, whereas spatial gradient reorientation is analogous to gradient-descent-based optimization. Gradient descent is commonly used for convex optimization; however, it is notoriously ineffective for global optimization and for optimization in non-convex settings, when compared with Langevin diffusion (Ma et al., 2019; Raginsky et al., 2017; Xu et al., 2018). Comparing trade-offs between sampling and optimization may therefore help understand the navigational strategies employed by organisms.

### Limitations of the study

In this study we propose a function for temporal fluctuations in pathway gain—they allow bacteria to effectively balance localization around peaks with navigation to new peaks. It will be important to test this proposal experimentally. In particular it will be important to experimentally quantify the navigation behavior of bacteria in multi-modal environments and how it is influenced by the pathway gain of individual bacteria.

## STAR★Methods

### Key resources table

**Table:** 

REAGENT or RESOURCE	SOURCE	IDENTIFIER
**Software and algorithms**		

Mathematica	https://www.wolfram.com/mathematica/	Version 12.1.1.0

### Resource availability

#### Lead contact

Further information and requests should be directed to and will be fulfilled by the lead contact, Uri Alon (uri.alon@weizmann.ac.il).

#### Materials availability

The study did not generate any new materials.

#### Data and code availability

• All data generated are available from the authors upon request.
• Code for the patch colonization simulations (Figures 5 and S4) is available at https://github.com/omerka-weizmann/chemotaxis_tempering.
• No additional information.

### Experimental model and subject details

The study did not use experimental models.

### Method details

#### Stochastic simulations of chemotaxis

We simulated the trajectories of bacteria using the Langevin process dx=χ ∇logL(x)dt+σdW, with $\sigma =\sqrt{2}\cdot 0.03\;mm^{1.5}sec^{-0.5}$ (corresponding to a run duration of τ=1secand a swimming speed of v=0.03mm/sec, and $D=v^{2}\tau$). The advection parameter χ was adjusted according to β, where $\beta =2\text{χ}/\sigma^{2}$. All simulations were performed using the ItoProcess procedure of Mathematica, with a step size of 0.05sec. For tempering between $\beta_{hot},\;\beta_{cold}$, we assumed stochastic changes with transition rates: $\eta_{1}:\beta_{cold}\to \;\beta_{hot},\;\eta_{2}:\beta_{hot}\to \;\beta_{cold}$, so transition that times were drawn from exponential distributions with means $\tau_{1}=\left(1/\eta_{1}\right),\tau_{2}=\left(1/\eta_{2}\right)$. Since stochastic fluctuations may occur on a timescale of minutes to hours, we chose for the simulations $\tau_{1}=6\;\text{min}$ and $\tau_{2}=24\text{ min}$. The generation interval is $t_{gen}=60\;\text{min}$.

#### Heterogeneity in β from bacterial T-maze experiments

A recent study (Salek et al., 2019) quantified non-genetic variability in pathway gain. While the fitted distribution is a product of several underlying components, we find that it is approximately a Gamma distribution with a shape parameter of 2.25 and a scale parameter of 35. This distribution has a coefficient of variation of approximately 2/3. We accounted for this heterogeneity in Figure 2 by computing the probability distribution $P\left(x\right)=\frac{1}{Z}\overset{\infty }{\underset{0}{\int }}Prob\left[\xi \right]\frac{L{\left(x\right)}^{\beta \xi }}{\int_{-\infty }^{\infty }L{\left(x\right)}^{\beta \xi }dx}d\xi$, where *Z* is a normalization factor, ξ is a random variable that is distributed ξ∼Gamma[2.25,35], and β is chosen such that <β⋅ξ>=14.

#### Adaptation time of tumbling rate

To derive the adaptation time for the tumbling rate *a* we linearize Equations 1 and 2 around $a=a_{0}$ and $m=m_{0}=1+\frac{1}{\alpha N}\log\frac{a_{0}}{1-a_{0}}+\frac{1}{\alpha }\log\frac{L}{K_{I}}$. Since tumbling rate adjusts on a timescale that is more rapid than methylation, we take its quasi-steady-state (given by Equation 1), so the equation for *m* becomes:

$$\dot{m}\left(t\right)\approx -\left(1-a_{0}\right)N\alpha \omega \left(m-\;m_{0}\right)\;$$

From which we can see that the adaptation rate is inversely proportional to $\left(1-a_{0}\right)N\alpha \omega$.

#### Mixing time of chemotaxis strategies

In order to analyze whether a given chemotaxis strategy is able to cross between peaks, we use a formal analogy between chemotaxis and the LMC algorithm. The LMC algorithm samples an invariant probability distribution that is proportional to the attractant distribution raised to the power β, where β is the inverse temperature of the Langevin process. Whether the chemotaxis strategy is able to cross between modes and localize around attractant peaks is then directly related to the convergence rate of the analogous sampling algorithm to its invariant distribution.

To quantify this convergence rate of the sampling process, we recall that we defined the KL divergence of the sampled distribution $\rho_{t}$ relative to the invariant distribution $l_{\beta }$:

$$H_{l_{\beta }}\left(\rho_{t}\right)=\int \rho_{t}\left(x\right)\log\frac{\rho \left(x\right)}{l_{\beta }\left(x\right)}dx$$

And that $H_{l_{\beta }}\left(\rho_{t}\right)\to 0$ as t→∞. We define the *mixing time* as the time it takes for $H_{l_{\beta }}\left(\rho_{t}\right)$ to reach a fraction γ of its original value, e.g. the half-way convergence point for γ=1/2. In our case, $l_{\beta }\left(x\right)$ is given by a power β of the attractant ligand distribution. As we have shown in the main text, for an Α log-concave attractant distribution such as a Gaussian $l_{\beta }\left(x\right)=e^{-\frac{\beta }{2\sigma^{2}}x^{2}\;}$ (which is log concave with $\text{Α}=\beta /\sigma^{2}$), the convergence rate can be bound by:

$$H_{l_{\beta }}\left(\rho_{t}\right)\leq e^{-2\alpha t}H_{l_{\beta }}\left(\rho_{0}\right)$$

And therefore, the mixing time is polynomial in the length-scale σ.

For the case of two or more Gaussians with means placed at distance 2μ from each other however, the limiting timescale for the mixing time is given by the escape time from each Gaussian, $\tau_{escape}\approx {\left(\frac{\text{βD}\sqrt{\mu^{2}-\sigma^{2}}}{2\pi \sigma^{3}}2^{\beta }\right)}^{-1}e^{\frac{\mu^{2}}{2\sigma^{2}}\beta }$, which is exponential in the length scale.

What about a tempered strategy? Here we will outline the proof of (Lee et al., 2018) that a tempered strategy over Gaussians of similar variance has polynomial mixing time. For simplicity, we analyze the discrete time case, as the results extend in a straightforward manner to the continuous time case. Following the definitions in (Madras and Randall, 2002; Lee et al., 2018), a Markov Process M is given by a probability transition kernel P(x,dy) over a measure space Ω (e.g. $\text{Ω}=R^{\text{n}}$), that is reversible with respect to a probability density π. The asymptotic convergence rate is related to a quantity that is called the *spectral gap* of the process. The spectral gap is defined as:

$$Gap\left(M\right)=\underset{\text{f}}{\inf}\frac{1}{2}\iint {\left|f\left(x\right)-f\left(y\right)\right|}^{2}\pi \left(dx\right)P\left(x,dy\right)$$

Where f is a non-constant function with variance 1 and an expected value of 0 w.r.t π. Note that the quantity that is minimized is the *Dirichlet form* of f.

The spectral gap is interesting because it determines the mixing time (Levin and Peres, 2017). The mixing time is inversely related to the spectral gap, so algorithmic improvement is achieved by increasing the spectral gap.

In our case, we will be interested in giving lower bounds on the spectral gap for Langevin diffusion processes on Gaussian patches, using a decomposability theorem, due to Randal and Madras (Madras and Randall, 2002).

For the decomposability theorem, we assume that we are given a partition $\text{Φ}=\left\{A_{j},\;j\in 1,..,m\right\}$ of the state space Ω of the Markov chain $\text{Ω}=\cup_{1\leq j\leq m}A_{j}$. Let $P{|}_{A}$ denote the restriction of P to A, so transitions occur according to P but are rejected if they leave A. Additionally, let $\bar{M}$ denote the projected Markov chain with transition rates corresponding to average probability flows between the sets of Φ, i.e. the transition probability between *i,j* is $\frac{1}{\pi \left(A_{i}\right)}\underset{A_{i}}{\int }\underset{A_{j}}{\int }P\left(x,dy\right)\pi \left(x\right)dx$. Then the decomposability theorem states the following lower bound on the spectral gap:

$$GAP\left(M\right)\geq \frac{1}{2}GAP\left(\bar{M}\right)\min_{1\leq \text{j}\leq \text{m}}GAP\left({M|}_{A_{j}}\right)$$

The product is composed of two parts: the spectral gap of the projected Markov chain $GAP\left(\bar{M}\right)$, and the smallest spectral gap when the Markov process is restricted to each component of the partition. More recently, Lee et al. demonstrated a similar decomposition theorem, which concerned decomposing the stationary distribution instead of decomposing the state space (Lee et al., 2018). For a Langevin-diffusion process over a mixture of Gaussians of similar variance, the stationary distribution is similar to a mixture of Gaussians (raised by a power β), over each of which the Langevin process mixes rapidly (Bakry and Émery, 1985), so the partition components can simply be chosen to be the mixture components. In fact, this can be generalized to any mixture of log-concave distributions (Lee et al., 2018).

Let us now consider a tempering strategy as defined in the manuscript, which consists of stochastic switches between two inverse temperatures $\beta_{cold},\beta_{hot}$ at rates $\eta_{1}:\beta_{cold}\to \;\beta_{hot},\;\eta_{2}:\beta_{hot}\to \;\beta_{cold}$. If $\beta_{hot}$ is chosen low enough then all the mixture components will become sufficiently close to allow for rapid transitions between the components at $\beta_{hot}$. It is therefore possible for the process to cross between the components at $\beta_{cold}$ by transitioning to $\beta_{hot}$ and then back to $\beta_{cold}$.

### Quantification and statistical analysis

#### Estimating the inverse temperature β from chemotaxis in linear gradients

To quantify the inverse temperature parameter for *E. coli* chemotaxis, we used the experiments by (Kalinin et al., 2009) that measured population distributions F(x) for *E. coli* in linear gradients after reaching dynamic equilibrium (Figure 5, panel E1, see Kalinin et al. for experimental details). In order to determine β, we estimated the following error: the absolute difference between the probability distribution proportional to $P\left(x\right)=L{\left(x\right)}^{\beta }$ (where L(x) is ligand concentration) and F(x) for each of the three gradients. We set β to minimize the average error for the three distributions. A similar fit can be obtained by taking the intercept of the linear fit of logF(x) against logL(x).
