# Supplementary material for: Temporal fluctuations in chemotaxis gain implement a simulated-tempering strategy for efficient navigation in complex environments
Source: iScience. 2021 Jun 28;24(7):102796. doi: 10.1016/j.isci.2021.102796 (PMC8319753; doi:10.1016/j.isci.2021.102796)
Supplement: Document S1. Figures S1–S4 and Table S1 [file mmc1.pdf]

iScience, Volume *24*

Supplemental information

Temporal fluctuations in chemotaxis gain implement a simulated-tempering strategy for efficient navigation in complex environments

Omer Karin and Uri Alon

Figure S1. Stochastic tempering for gradients that decay like 1/x. Related to Figure 3.

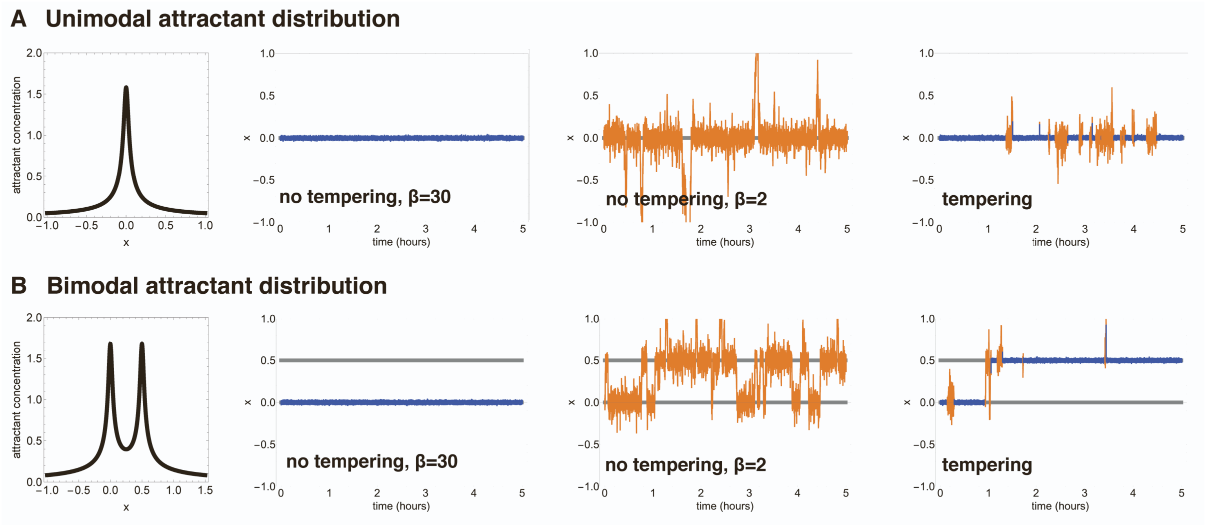

Chemotaxis simulations were performed as in Figure 3 with either unimodal (A) or bimodal (B) distributions. Here patches have the form $\frac{1}{\sqrt{\ell+(x-\mu)^2}}$ (taking $\ell = 10^{-3}$) which decay like $\frac{1}{x}$ but have a continuous logarithmic derivative at $x = \mu$. The distributions are proportional to $L(x) = \frac{1}{\sqrt{\ell+x^2}}$ (unimodal) or $L(x) = \frac{1}{\sqrt{\ell+x^2}} + \frac{1}{\sqrt{\ell+(x-0.5)^2}}$ (bimodal). As in the case of Gaussian patches, high β provides tight accumulation around attractant peaks but fails to cross between peaks, whereas low β facilitates crossing between peaks but provides poor accumulation around peaks. Tempering allows to balance tight accumulation around peaks with efficient crossing between peaks.

Figure S2. Dependence of escape time on $\boldsymbol{\beta}$ and environmental parameters. Related to Figure 4.

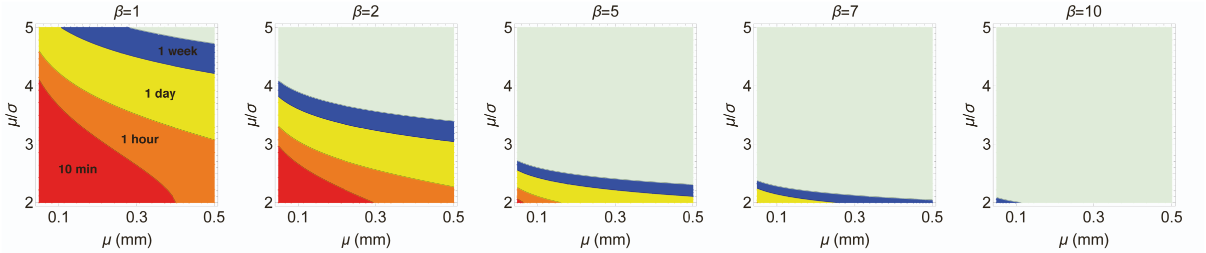

Contour plot showing τ_{escape} estimated from Eq. 7, for various values of μ, σ, β (left to right: $\beta = 1,2,5,7,10$). The contours are $\tau_{escape} < 10\,\mathrm{min}$ (red), $\tau_{escape} < 1\,\mathrm{hour}$ (orange), $\tau_{escape} < 1\,\mathrm{day}$ (yellow), $\tau_{escape} < 1\,\mathrm{week}$ (blue), $\tau_{escape} > 1\,\mathrm{week}$ (light green).

Figure S3. Patch escape time and performance for different patch lifetimes. Related to Figure 4.

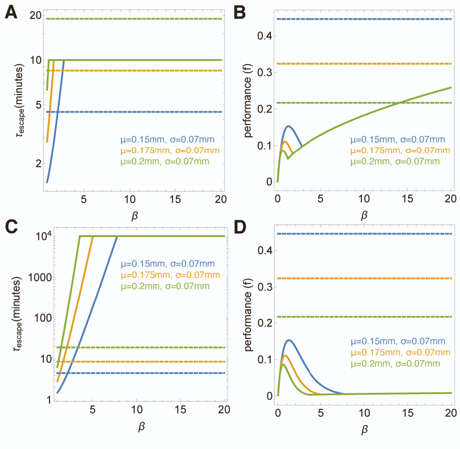

Time to cross over to an adjacent patch τ_{escape}, and performance f (as in Figure 4BC) plotted for a patch lifetime of $\tau_{patch} = 10\text{min}$ (panels AB) or $\tau_{patch} = 1\text{week}$ (panels CD). Solid lines correspond to the untempered strategy, while dashed lines correspond to the tempered strategy plotted for $\beta_{hot} = 1, \beta_{cold} = 20, \epsilon = \frac{1}{3}$. All other parameters are as in Figure 4BC.

Figure S4. Patch colonization for different levels of metastability. Related to Figure 5.

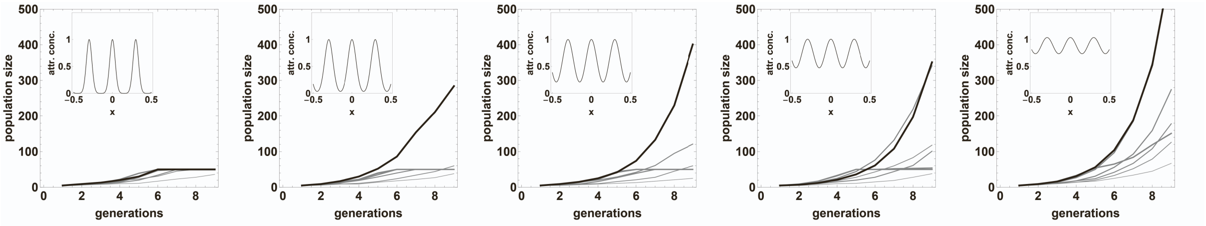

To test the sensitivity of the tempered strategy to the degree of metastability, we repeated the simulations of Figure 5 and adjusted the standard deviation parameter σ (Figure S3, from left to right: $\sigma = 0.035mm, \sigma = 0.053mm, \sigma = 0.07mm, \sigma = 0.088mm, \sigma = 0.11mm$. All other parameters are the same as in Figure 5. The absolute values were taken to be much larger than the typical run length of *E. coli*.

The lowest σ has the highest degree of metastability – the modes are separated to the extent that even the $\beta = \beta_{hot}$ strategy mixes poorly. This case is then effectively like the unimodal case and therefore the optimal strategy maximizes β. However, since the tempered strategy spends only a small portion of the time in low β, it still performs similarly to the optimal untempered strategy.

For the highest σ cases, the modes are hardly separated, so even a high β strategy can cross between them, although at very high β metastability will still prevent crossing between modes. In this case, again, the tempered strategy again performs similarly to the optimal untempered strategy, without needing to tune β.

Finally, for the cases of intermediate separation of modes, the tempered strategy outperforms the untempered strategies, as discussed in Figure 5. We therefore conclude that the tempering strategy is robust for the patch colonization task – it performs similarly to the optimal untempered strategy for environments where metastability is not important, and outperforms all untempered strategies when metastability is important.

Table S1. Estimates for parameter values. Related to Figure 2.

Parameter	Value
α	1.7
N	6
a_0	0.5
K_I	18.2 μM
K_A	3 mM
ω	0.005 s^{-1}
H	10
z_θ	0.14 s^{-1}
τ	0.8 s

All values are taken from Si et al. 2012.
